# Socioeconomic status and self-reported asthma in Indigenous and non-Indigenous Australian adults aged 18-64 years: analysis of national survey data

**DOI:** 10.1186/1475-9276-9-18

**Published:** 2010-08-10

**Authors:** Joan Cunningham

**Affiliations:** 1 Menzies School of Health Research, Charles Darwin University, PO Box 41096, Casuarina, Northern Territory 0811, Australia

## Abstract

**Background:**

Asthma is more common among Indigenous than non-Indigenous Australian adults, but little is known about socioeconomic patterning of asthma within the Indigenous population, or whether it is similar to the non-Indigenous population.

**Methods:**

I analysed weighted data on self-reported current diagnosed asthma and a range of socio-economic and demographic measures for 5,417 Indigenous and 15,432 non-Indigenous adults aged 18-64 years from two nationally representative surveys conducted in parallel by the Australian Bureau of Statistics in 2004-05.

**Results:**

Current asthma prevalence was higher for Indigenous than non-Indigenous people in every age group. After adjusting for age and sex, main language and place of residence were significantly associated with asthma prevalence in both populations. Traditional SES variables such as education, income and employment status were significantly associated with asthma in the non-Indigenous but not the Indigenous population. For example, age-and sex-adjusted relative odds of asthma among those who did not complete Year 10 (versus those who did) was 1.2 (95% confidence interval (CI) 1.0-1.5) in the non-Indigenous population versus 1.0 (95% CI 0.8-1.3) in the Indigenous population.

**Conclusions:**

The socioeconomic patterning of asthma among Indigenous Australians is much less pronounced than for other chronic diseases such as diabetes and kidney disease, and contrasts with asthma patterns in the non-Indigenous population. This may be due in part to the episodic nature of asthma, and the well-known challenges in diagnosing it, especially among people with limited health literacy and/or limited access to health care, both of which are more likely in the Indigenous population. It may also reflect the importance of exposures occurring across the socioeconomic spectrum among Indigenous Australians, such as racism, and discrimination, marginalization and dispossession, chronic stress and exposure to violence.

## Background

Despite its status as an important cause of morbidity worldwide, the epidemiology of asthma remains less developed than that of other chronic conditions such as heart disease and cancer [1,2]. In recent years, there has been increasing recognition of the importance of the social as well as physical environment in the development of asthma [3]. A range of social factors have been of interest, with socioeconomic status (SES) and ethnicity receiving perhaps the most widespread attention to date. Although most studies of SES and asthma have focused on childhood asthma, several studies have found significant associations between various measures of SES and asthma in adults [4-7].

Australia has a high asthma prevalence by world standards [2], and asthma has been designated a National Health Priority Area [8]. Asthma is more common among Indigenous than non-Indigenous adults in Australia [8], but little is known about the distribution of asthma *within *the Indigenous population. Recent studies have found significant inverse socioeconomic gradients in both end-stage kidney disease and diabetes among Indigenous Australians [9-12], but it is unclear whether this applies to other chronic conditions such as asthma, or whether any socioeconomic gradients in the Indigenous population are of similar magnitude to those in the non-Indigenous population.

The aim of the current study is to examine the relationships between indicators of socioeconomic status and self-reported asthma among a nationally representative sample of Indigenous Australian adults, and to compare these with corresponding patterns in the non-Indigenous population.

## Methods

Data for Indigenous and non-Indigenous adults aged 18-64 years were taken from two national surveys conducted in parallel by the Australian Bureau of Statistics (ABS) in 2004-05: the National Aboriginal and Torres Strait Islander Health Survey (NATSIHS) and the National Health Survey (NHS). These two surveys had very similar content and in most cases the wording of questions on particular topics was identical [13]. This analysis is limited to responses to questions deemed by the ABS to be comparable in the two surveys [14].

Extensive details on survey methodology have been published elsewhere [13-18]. Briefly, both surveys were conducted using multi-stage sampling strategies; the first stage involved random selection of either communities or census collection districts (CD), and subsequent stages involved selection of dwellings and individuals within households [15,18]. Indigenous respondents from the NHS were included with NATSIHS data to provide Indigenous population estimates [15]. Both surveys were limited to usual residents of private dwellings and conducted by trained ABS interviewers. Very remote areas were out of scope in the NHS but not the NATSIHS. In the NHS and in non-remote areas in the NATSIHS, data were collected using Computer Assisted Interviews. In remote areas of the NATSIHS, pen and paper interview forms were used and some questions were simplified or deleted. More details about the design, conduct and results of the surveys are available elsewhere [13-18].

To allow data access to interested researchers, the ABS created a Confidentialised Unit Record File (CURF) for the NATSIHS. This file includes unit records for Indigenous respondents of the 2004-05 NATSIHS and the 2004-05 NHS, as well as unit records for non-Indigenous respondents from the 2004-05 NHS [14]. Although the CURF contains unit records for participants of all ages, this analysis is limited to data from the 20,849 adult respondents (5,417 Indigenous and 15,432 non-Indigenous) aged 18-64 years. The exclusion of those aged ≥65 years was due to uncertainty about the applicability of socioeconomic indicators among older people, as well as the relatively small size of this group in the Indigenous population [19]. Children were excluded because information was not available/not relevant for most SES indicators.

### Definition of asthma

Participants were asked whether they had ever been told by a doctor or nurse that they had asthma and, if so, whether they still had asthma. For the purposes of this analysis, asthma was considered to be present if the participant responded positively to both questions.

### Socio-demographic factors

Information was available on a range of socioeconomic and demographic factors, as shown in Table 1. Information about age and sex of household members, and whether the respondent was currently attending school was provided by 'any responsible adult' within the household; information about the dwelling and the income of non-participant household members (required to calculate household income) was provided by a household 'spokesperson', chosen on the basis of his or her ability to provide accurate information. Information relating to geography (including remoteness classification and area-level disadvantage score) was provided by the ABS based on the CD in which the selected dwelling was located. All other information used in this analysis was provided by the respondent [15].

**Table 1:** Socio-demographic characteristics of Indigenous and non-Indigenous Australians aged 18-64 years, 2004-05.*

	Indigenous % (95% CI)†	Non-Indigenous % (95% CI)†
Age (years)		
18-24	23.1 (21.7-24.4)	15.1 (14.8-15.4)
25-34	28.4 (27.7-29.0)	22.4 (22.3-22.6)
35-44	24.0 (23.5-24.5)	23.5 (23.4-23.7)
45-54	16.1 (15.7-16.4)	22.0 (21.8-22.1)
55-64	8.5 (7.1-9.9)	17.0 (16.9-17.1)
Sex		
Male	46.8 (45.6-47.9)	49.8 (49.6-50.1)
Female	53.2 (52.1-54.4)	50.2 (49.9-50.4)
Main language spoken at home		
English	86.0 (84.5-87.5)	90.8 (89.9-91.7)
Not English	14.0 (12.5-15.5)	9.2 (8.3-10.1)
Highest year of school completed		
Year 12	23.5 (21.2-25.8)	52.5 (51.2-53.8)
Year 11	13.0 (11.7-14.4)	10.9 (10.3-11.6)
Year 10	31.2 (29.4-33.1)	24.7 (23.7-25.7)
Year 9	13.9 (12.5-15.3)	6.3 (5.8-6.7)
≤Year 8 or never went to school	18.3 (16.7-20.0)	5.6 (5.1-6.1)
Level of highest non-school qualification		
Post-graduate degree	1.9 (1.0-2.7)	6.2 (5.8-6.7)
Bachelor's degree	2.9 (2.3-3.6)	14.5 (13.8-15.3)
Diploma	4.7 (3.7-5.7)	9.7 (9.1-10.3)
Certificate	24.2 (22.2-26.1)	26.0 (25.0-27.1)
No qualifications	66.4 (64.1-68.6)	43.5 (42.5-44.5)
Employment status		
Employed	54.7 (52.2-57.1)	76.1 (75.3-76.8)
Unemployed	8.1 (6.9-9.2)	3.0 (2.7-3.4)
Not in the labour force	37.3 (35.0-39.6)	20.9 (20.1-21.7)
Housing tenure		
Owner/purchaser‡	24.7 (22.1-27.3)	n/a§
Renter/other tenure	75.3 (72.7-77.9)	n/a§
Equivalised household income quintile||		
1 (lowest)	33.7 (31.4-36.1)	11.3 (10.7-11.9)
2	21.6 (19.7-23.6)	13.1 (12.5-13.8)
3	14.3 (12.4-16.1)	16.9 (16.1-17.6)
4	9.4 (7.7-11.2)	19.5 (18.7-20.2)
5 (highest)	5.2 (4.0-6.4)	21.7 (20.7-22.7)
Not known or not stated	15.6 (13.6-17.6)	17.5 (16.6-18.4)
Reported food insecurity**		
Yes	24.6 (22.7-26.6)	5.6 (5.1-6.0)
No	75.4 (73.4-77.3)	94.4 (94.0-94.9)
SEIFA quintile††		
1 (most disadvantaged)	49.3 (43.7-55.0)	17.1 (15.7-18.5)
2	19.3 (15.2-23.3)	19.0 (17.4-20.7)
3	18.5 (14.3-22.7)	20.3 (18.4-22.2)
4	9.0 (6.4-11.6)	21.3 (19.5-23.0)
5 (least disadvantaged)	3.9 (2.2-5.7)	22.3 (20.0-24.7)
Area of residence‡‡		
Major cities	30.6 (29.1-32.0)	70.2 (68.6-71.8)
Inner regional	20.1 (19.0-21.3)	19.5 (17.9-21.0)
Outer regional	21.5 (20.4-22.5)	10.4 (9.2-11.5)
Remote or very remote	27.8 (26.3-29.4)	---§§
Smoking status		
Current smoker	53.5 (51.2-55.8)	25.7 (24.8-26.7)
Former smoker	16.8 (15.1-18.5)	23.2 (22.4-24.1)
Never smoker	29.7 (27.6-31.9)	51.0 (49.7-52.3)

* Weighted data from the National Aboriginal and Torres Strait Islander Health Survey 2004-05 confidentialised unit record file (CURF) [14].

† CI, confidence interval. Proportions are weighted to provide population estimates. Totals are based on those with non-missing data, except for equivalised household income, for which a separate category is shown.

‡ Includes ownership/purchase by any of the dwelling's occupants (not necessarily the respondent) [15,17].

§ n/a, not available.

|| Gross weekly equivalised cash income of household, which takes into account household size and composition, was estimated using the OECD scale [15,17]. Quintiles were determined based on all-Australian figures. That is, the same category boundaries were used for both Indigenous and non-Indigenous participants.

** Food insecurity was based on the response to a question about whether, in the past 12 months, the respondent had run out of food and couldn't afford to buy more [15].

†† SEIFA, Socioeconomic Index for Areas, Index of Relative Disadvantage. SEIFA quintile was based on the 2001 score for the CD of the selected dwelling and is used as a measure of area-level disadvantage [15,17]. Quintiles were determined based on all-Australian figures. That is, the same category boundaries were used for both Indigenous and non-Indigenous respondents.

‡‡ Classified according to the Australian Standard Geographical Classification remoteness classification (based on the ARIA+ index) [15,17].

§§ Out of scope - not included in the survey [14,15].

### Statistical analysis

All analyses were conducted using STATA version 10.0 via the ABS's Remote Access Data Laboratory (RADL). Under the RADL system, analysts submit statistical code to the ABS; the code is then run and the output made available to the analyst through a password-protected web account. Analysts do not have direct access to unit record data at any time, and there are limits placed on the commands and outputs that are allowed, in order to protect the security and confidentiality of the data [20].

All analyses used ABS-generated person-weights (or expansion factors) to adjust for disproportionate sampling of some groups. The estimates produced in this manner apply to the population as a whole, and not just the sample [14,21]. Standard errors and 95% confidence intervals (CI) were calculated using replicate weights produced by the ABS using the Jackknife method (250 replicate weights for Indigenous respondents, 60 for non-Indigenous respondents) [14,21]. These replicate weights allow estimation of standard errors taking into account the complex design and weighting procedures used in the surveys [15,21]. Although STATA version 10 incorporates a suite of procedures to analyse complex survey data, these commands are not allowed in the RADL system (Therese Lalor, ABS, personal communication, May 2009). Instead, commands from the *svr *module written by Nick Winter (available using the STATA command: search svr, net) were used.

Directly age-standardised estimates and 95% CIs were calculated using an alternative set of person-weights and replicate weights produced by ABS for that purpose. The standard population was the total Australian population as at 30 June 2001 [14].

Logistic regression was conducted separately for Indigenous and non-Indigenous groups due to the different numbers of replicate weights for the two groups. All models were adjusted for age group and sex, with socioeconomic variables assessed individually and in combination. Participants with missing data were excluded only from analyses involving the variable for which they were missing data. The proportion of participants with missing data was small for all variables with the exception of equivalised household income quintile, which was not available for 2,941 respondents (14.1% overall, including 14.4% of Indigenous respondents and 14.0% of non-Indigenous respondents). Analyses were conducted with these respondents coded as missing, as well as with them included using a special category of household income unknown.

### Ethics approval

This study was approved by both the Aboriginal sub-committee and the main committee of the Human Research Ethics Committee of the Northern Territory Department of Health and Families and Menzies School of Health Research.

## Results

Over 1 in 4 Indigenous people (27.5%, 95% CI 25.5-29.5) aged 18-64 reported they had ever been diagnosed with asthma, and 1 in 6 (16.2%, 95% CI 14.6-17.8) said they currently had asthma. The corresponding figures were significantly lower in the non-Indigenous population: ever asthma 20.6% (95% CI 19.7-21.5); current asthma 9.9% (95% CI 9.3-10.4).

**Figure 1:**
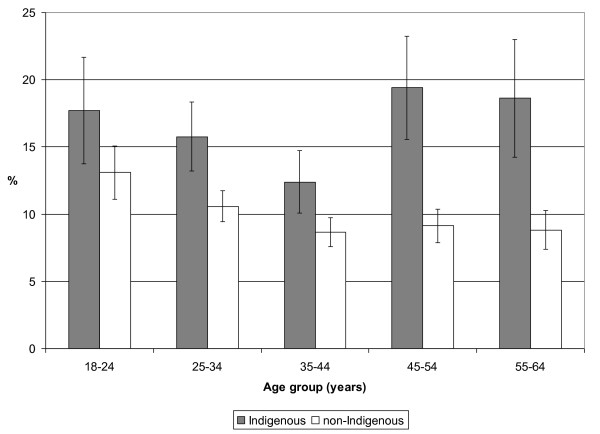
**Prevalence (% and 95% confidence interval) of self-reported current asthma by age and Indigenous status, Australian adults, 2004-05**. Weighted data from the National Aboriginal and Torres Strait Islander Health Survey 2004-05 confidentialised unit record file [14].

Current diagnosed asthma was more commonly reported for Indigenous than non-Indigenous people in every age group (Figure 1). Asthma decreased with age until age 45 years in both groups. After age 45, it leveled off in the non-Indigenous population but increased in the Indigenous population (Figure 1). As a result, the Indigenous: non-Indigenous prevalence ratio increased from about 1.3-1.5 in younger age groups to 2.1 in older age groups.

The socio-demographic profile of the Indigenous population was significantly different from that of the non-Indigenous population, with a younger age distribution, lower educational attainment, and greater levels of disadvantage across a range of indicators (Table 1).

**Figure 2:**
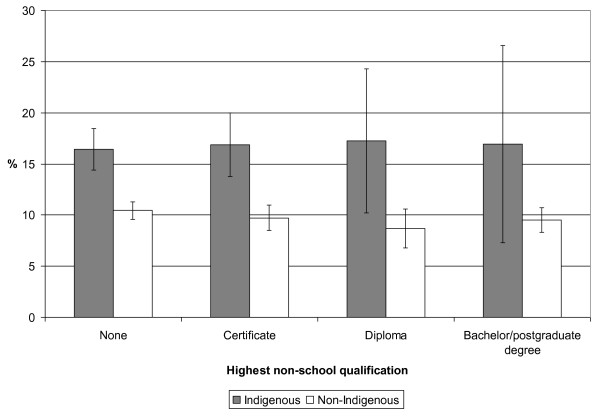
**Age-standardised prevalence (% and 95% confidence interval) of self-reported current asthma by highest non-school qualification for Indigenous and non-Indigenous Australian adults, 2004-05**. Weighted data from the National Aboriginal and Torres Strait Islander Health Survey 2004-05 confidentialised unit record file [14].

**Figure 3:**
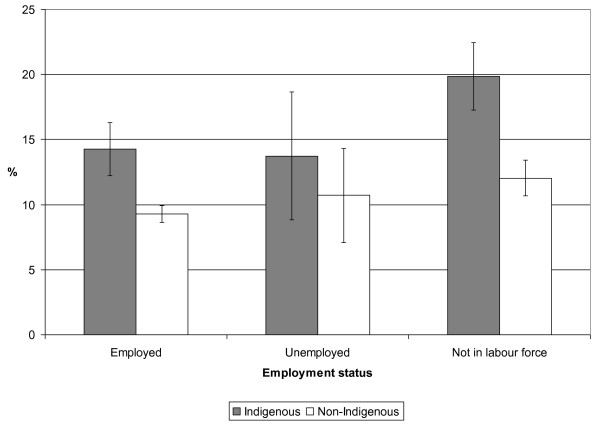
**Age-standardised prevalence (% and 95% confidence interval) of self-reported current asthma by employment status for Indigenous and non-Indigenous Australian adults, 2004-05**. Weighted data from the National Aboriginal and Torres Strait Islander Health Survey 2004-05 confidentialised unit record file [14].

Age-standardised asthma prevalence was higher for Indigenous people than non-Indigenous people of the same SES category across every variable examined (see Figures 2 and 3, for example).

After adjusting for age and sex, self-reported asthma was significantly lower among Indigenous and non-Indigenous people whose main language was not English (Table 2). Compared to their peers in major cities, asthma was significantly less common among Indigenous people in remote areas, and significantly more common among non-Indigenous people in inner regional areas (Table 2). Main language and place of residence were associated with asthma independent of one another in both populations (data not shown).

**Table 2:** Relative odds of current asthma by socioeconomic status variables for Indigenous and non-Indigenous Australians aged 18-64 years, 2004-05.*

	Indigenous		
	**Adjusted for age and sex** **OR (95% CI)†**	**Adjusted for age, sex, main language and area of residence** **OR (95% CI)†**	**Full model‡** **OR (95% CI)†**

Main language			
English	1.0	---	1.0
Not English	**0.4 (0.3-0.6)**	---	**0.5 (0.3-0.8)**
Highest year of school completed			
Year 10 or more	1.0	1.0	1.0
Less than Year 10§	1.0 (0.8-1.3)	1.1 (0.9-1.4)	1.0 (0.8-1.3)
Highest non-school qualification			
Bachelor/post-graduate degree	1.0 (0.5-1.9)	0.9 (0.5-1.6)	0.9 (0.4-1.7)
Diploma	1.1 (0.7-2.0)	1.0 (0.6-1.7)	1.0 (0.5-1.8)
Certificate	1.2 (0.9-1.6)	1.1 (0.9-1.5)	1.1 (0.8-1.6)
No qualifications	1.0	1.0	1.0
Employment status			
Employed	1.0	1.0	1.0
Unemployed	0.9 (0.6-1.5)	0.9 (0.6-1.5)	0.9 (0.5-1.5)
Not in the labour force	1.0 (0.8-1.3)	1.1 (0.8-1.4)	1.0 (0.7-1.3)
Housing tenure			
Owner/purchaser	1.1 (0.8-1.5)	0.9 (0.7-1.2)	1.0 (0.7-1.3)
Renter/other tenure	1.0	1.0	1.0
Ran out of food and couldn't afford to buy more (last 12 mos)			
Yes	1.2 (0.9-1.4)	**1.3 (1.1-1.7)**	**1.3 (1.0-1.7)**
No	1.0	1.0	1.0
Equivalised household income quintile||			
1 (lowest)	0.7 (0.4-1.3)	0.8 (0.5-1.5)	0.7 (0.3-1.4)
2	0.6 (0.3-1.1)	0.7 (0.4-1.3)	0.6 (0.3-1.2)
3	0.7 (0.4-1.4)	0.8 (0.4-1.4)	0.7 (0.3-1.4)
4	0.6 (0.3-1.3)	0.6 (0.3-1.3)	0.6 (0.3-1.3)
5 (highest)	1.0	1.0	1.0
Not known/not stated	0.6 (0.3-1.2)	0.8 (0.4-1.4)	0.7 (0.3-1.5)
SEIFA quintile††			
1 (most disadvantaged)	1.5 (0.4-6.3)	2.1 (0.5-9.3)	2.0 (0.5-8.2)
2	1.8 (0.4-7.5)	2.1 (0.5-9.3)	2.0 (0.5-8.6)
3	1.7 (0.4-7.4)	2.0 (0.5-9.2)	2.0 (0.5-8.4)
4	1.6 (0.4-6.8)	1.8 (0.4-7.9)	1.8 (0.4-7.5)
5 (least disadvantaged)	1.0	1.0	1.0
Area of residence			
Major cities	1.0	---	1.0
Inner regional	1.0 (0.7-1.4)	---	0.9 (0.7-1.3)
Outer regional	0.8 (0.5-1.1)	---	0.8 (0.5-1.1)
Remote or very remote	**0.4 (0.3-0.6)**	---	**0.5 (0.3-0.7)**
			

	**Non-Indigenous**		

	**Adjusted for age and sex OR (95% CI)†**	**Adjusted for age, sex, main language and area of residence OR (95% CI)†**	**Full model‡ OR (95% CI)†**

Main language			
English	1.0	---	1.0
Not English	**0.3 (0.2-0.4)**	---	**0.3 (0.2-0.4)**
Highest year of school completed			
Year 10 or more	1.0	1.0	1.0
Less than Year 10§	**1.2 (1.0-1.5)**	**1.3 (1.1-1.6)**	1.2 (1.0-1.5)
Level of highest non-school qualification			
Bachelor/post-graduate degree	0.9 (0.8-1.1)	1.0 (0.8-1.1)	1.1 (0.9-1.3)
Diploma	0.8 (0.6-1.1)	0.8 (0.7-1.1)	1.0 (0.7-1.2)
Certificate	1.0 (0.8-1.2)	1.0 (0.8-1.1)	1.0 (0.9-1.2)
No qualifications	1.0	1.0	1.0
Employment status			
Employed	1.0	1.0	1.0
Unemployed	1.1 (0.7-1.6)	1.2 (0.8-1.7)	1.0 (0.6-1.5)
Not in the labour force	**1.3 (1.1-1.5)**	**1.4 (1.2-1.6)**	**1.3 (1.1-1.5)**
Housing tenure			
Owner/purchaser	---	---	---
Renter/other tenure	---	---	---
Ran out of food and couldn't afford to buy more in last 12 months			
Yes	**1.8 (1.5-2.3)**	**1.8 (1.4-2.2)**	**1.5 (1.2-2.0)**
No	1.0	1.0	1.0
Equivalised household income quintile||			
1 (lowest)	**1.3 (1.0-1.6)**	**1.4 (1.1-1.8)**	1.2 (0.9-1.5)
2	1.2 (0.9-1.5)	1.3 (1.0-1.6)	1.1 (0.9-1.5)
3	0.9 (0.7-1.1)	1.0 (0.8-1.2)	0.9 (0.7-1.2)
4	0.9 (0.8-1.1)	1.0 (0.8-1.2)	1.0 (0.8-1.2)
5 (highest)	1.0	1.0	1.0
Not known/not stated	0.8 (0.6-1.0)	0.8 (0.6-1.1)	0.8 (0.6-1.1)
SEIFA quintile††			
1 (most disadvantaged)	1.2 (0.9-1.5)	**1.3 (1.0-1.7)**	1.2 (0.9-1.5)
2	1.2 (1.0-1.5)	1.2 (1.0-1.5)	1.1 (0.9-1.4)
3	1.1 (0.9-1.3)	1.1 (0.9-1.3)	1.0 (0.8-1.3)
4	1.2 (0.9-1.5)	1.1 (0.9-1.4)	1.1 (0.9-1.4)
5 (least disadvantaged)	1.0	1.0	1.0
Area of residence			
Major cities	1.0	---	1.0
Inner regional	**1.3 (1.1-1.5)**	---	1.1 (1.0-1.3)
Outer regional	1.1 (0.9-1.3)	---	0.9 (0.7-1.2)
Remote or very remote	---	---	---

* Weighted data from the National Aboriginal and Torres Strait Islander Health Survey 2004-05 confidentialised unit record file (CURF) [14].

† OR, odds ratio; CI, confidence interval. Figures in bold are statistically significant at p < 0.05.

‡ Full model includes age group, sex and all other variables listed.

§ Includes those who never went to school.

|| Gross weekly equivalised cash income of household, using the OECD scale [15]. Quintiles are based on national figures.

** SEIFA, Socioeconomic Index for Areas, Index of Relative Disadvantage. Quintiles are based on national figures.

In the Indigenous population, with the exception of language and place of residence, most odds ratios were close to the null value (Table 2). Household income and SEIFA score were not significantly associated with asthma regardless of whether the comparison was of individual quintiles versus the highest quintile (as shown in Table 2), of quintiles 1-4 combined versus the highest quintile, or any other combination of quintiles (data not shown). Similar results were observed when the analysis was limited to Indigenous people in non-remote areas, with the exception of main language, which was no longer significant (data not shown).

In the non-Indigenous population, educational attainment, labour force status, household income and food insecurity were significantly associated with asthma, while non-school qualifications and area-level disadvantage (as estimated by SEIFA quintile) were not (Table 2).

Adjusting for main language and place of residence in addition to age and sex generally resulted in only marginal changes to the odds ratios for SES variables in both Indigenous and non-Indigenous groups, although food insecurity (Indigenous) and SEIFA quintile (non-Indigenous) were statistically significant after adjustment for these additional variables (Table 2). Similarly, there were generally only modest changes to the odds ratios in a fully adjusted model (Table 2).

A history of smoking was more common among Indigenous than non-Indigenous respondents (Table 1), and asthma was more commonly reported by current and former smokers than by never smokers (Indigenous: 17%, 17% and 14% respectively; non-Indigenous: 11%, 10% and 9% respectively). However, adjustment for smoking history did not appreciably alter the relationships between SES variables and asthma in either group (data not shown).

## Discussion

Asthma was more commonly reported by Indigenous than non-Indigenous Australians in this nationally representative study, but the prevalence of asthma was not associated with most traditional indicators of SES - education, employment, income, home ownership and area-level disadvantage - in the Indigenous population. In the non-Indigenous population, associations between these traditional SES indicators and asthma were generally significant, although modest in size. In both populations, main language, along with place of residence and food insecurity, appeared to be more strongly associated with current asthma than traditional SES indicators.

The lack of an association between traditional SES variables and asthma among Indigenous Australians contrasts sharply with results of previous studies of other chronic conditions such as diabetes and kidney disease. In two recent studies, diabetes prevalence was strongly inversely associated with a wide range of SES measures among Indigenous Australians [11,12]. The higher rates of diabetes among Indigenous Australians were not completely explained by their relative disadvantage, however, as Indigenous people of high SES still had higher rates of diabetes than did non-Indigenous people of low SES [12]. Similarly, Cass and colleagues showed a strong gradient in regional rates of Indigenous Australian end-stage renal disease according to an index of social disadvantage [9]. Even in the least disadvantaged regions, however, age- and sex-standardised incidence of end-stage renal disease was generally significantly higher for Indigenous Australians than for the total Australian population [9,10].

Few studies have focused on the relationship between SES and asthma in indigenous populations in other developed countries. In one large American study using 2004 data from the Behavioral Risk Factor Surveillance System (BRFSS), the higher prevalence of asthma among Native American adults compared with non-Hispanic Whites was due in large part to their lower SES [5]. Although direct comparison was not possible in the present study, age-adjusted asthma prevalence was higher for Indigenous than non-Indigenous people of the same SES category across all variables examined, which suggests that the higher asthma prevalence of Indigenous Australians is not explained by SES differences.

The results for the non-Indigenous population are largely consistent with studies from other populations in developed countries. Among adults in 24 US states in 2004, education, income and employment status were all independently associated with asthma prevalence [5]. Using US NHANES data for 2001-2004, another study found that males and females living below the poverty line were more likely to report current asthma than those living at or above it [7]. In an analysis of 2005 BRFSS data, low household income (<$25,000 versus ≥$50,000) was significantly associated with asthma [6]. In a study of adults aged 20-44 years in 32 study centres in Europe, North America and Australasia, low social class (based on occupation) and low age at completion of full-time studies were associated with current asthma prevalence after adjustment for other individual level factors, and area-level educational level was associated with asthma prevalence, regardless of atopic status [4]. In California, higher education was associated with higher levels of asthma with hay fever (a marker of atopic status), but lower levels of asthma without hay fever [22].

The significantly lower prevalence of self-reported asthma among Indigenous and non-Indigenous people whose main language was not English is consistent with the higher rates of asthma in English-speaking countries generally [2]. Language may be a marker for lower levels of exposure to asthma risk factors, lower genetic susceptibility, lower access to and/or use of health services that would result in a diagnosis of asthma, or a combination of these and other factors. Differences in access to and use of health services may also help explain the lower prevalence of asthma in Indigenous people living in remote areas. Although main language varied by place of residence, these two variables were independently associated with asthma prevalence.

Food insecurity was associated with asthma in both populations after adjustment for other factors including SES. Food insecurity, which was more common in lower SES groups but was reported across the SES spectrum, may be a more salient measure of financial stress than traditional SES measures. Although not directly comparable, the results are consistent with data from the 2004 and 2005 US BRFSS indicating a significantly higher prevalence of asthma, even after adjusting for SES, among those who reported they couldn't see a doctor because of cost in the past 12 months [5,6].

The main strengths of the current study are the use of nationally representative data, comparisons between Indigenous and non-Indigenous populations, and identical SES measures with comparable scales in the two populations. The main limitations relate to the cross-sectional nature of the study and the potential misclassification of asthma, SES and other relevant factors.

Because information on SES and asthma were collected at the same time, the temporal relationships between SES indicators and asthma are not always certain. For example, employment status may change as a result of having a serious chronic disease such as severe asthma. This may explain why asthma was more common among those not in the labour force, at least in the non-Indigenous population.

Although the definition of asthma was limited to those who said they had been diagnosed by a health practitioner, it is possible that some people who reported asthma did not actually have it, or did not currently have it, while others who did have asthma did not report it (in some cases because they had never received a diagnosis), or reported that it was not current. It is possible that the higher prevalence of asthma in the Indigenous population, particularly in older age groups, may be explained in part by misdiagnosis of other chronic respiratory diseases as 'asthma', but other factors, such as inadequate treatment and greater lifelong exposure to tobacco smoke and respiratory infections, are likely to play an important role [8]. Conversely, factors such as lack of access to and/or use of diagnostic services may have resulted in an under-estimate of asthma prevalence. If this was more common among those of low SES, it could explain, at least in part, the lack of observed associations between traditional SES variables and asthma in the Indigenous population. It is worth noting, however, that other factors likely to be associated with low SES, such as food insecurity, remote area residence, and speaking a language other than English, were all significantly associated with asthma.

Information used to determine SES may have been incorrectly reported by (or on behalf of) some participants, and only limited detail was available on the SES indicators examined here. Data on housing tenure was not available in the NATSIHS CURF for the non-Indigenous population. Despite the use of comparable scales, the equivalence of a given level of SES may not be guaranteed across individuals or population groups. For example, the meaning of a certain level of education may vary over time and place, and years of education do not necessarily reflect the quality of education received, nor its social or economic value [23,24]. Similarly, the use of SEIFA quintiles based on the whole population may not adequately capture the socioeconomic position of population subgroups such as Indigenous Australians [25]. No information was available about other potentially important SES measures, such as total household assets. An area-based measure of disadvantage was included, but no other information was available about neighbourhood/area characteristics. Although equivalised household income is intended to adjust for household size and economies of scale, the dynamic nature of Indigenous households [26] can make it difficult to assess both Indigenous household income and household size, both of which are required to calculate equivalised income. This analysis assessed the associations between *adult *SES and current asthma in *adults*. No information was available about childhood SES for adult participants, even though their asthma may have first occurred during childhood. The factors associated with asthma in children may be different from those in adults. Although information about asthma was available for participating children in the NATSIHS and NHS, there were few SES data available for those under 18 years.

No information was available about a number of other factors that can affect asthma risk, including air quality, occupational exposures, family history of asthma, childhood infections, domestic exposures such as mould and dust mites, passive smoking, diet, and access to care. Despite these limitations, the NATSIHS data provide the best available information on asthma in Indigenous Australian adults.

While traditional SES variables do not appear to explain the patterns of asthma in Indigenous Australians, other factors that operate across the socioeconomic spectrum, including racism and discrimination, marginalization and dispossession, chronic stress, and exposure to violence [3,27-29], may play a role in asthma expression through a range of plausible biological pathways [30]. The episodic nature of asthma, and the well-known challenges in diagnosing it, may also be important, especially among people with limited health literacy and/or limited access to health care, both of which are more likely in the Indigenous population.

## Conclusions

Asthma has generally not been viewed as a major health problem for Indigenous Australians [31]. While it is true that other chronic health conditions such as diabetes and heart disease account for a large share of morbidity and mortality in the Indigenous population [19], the nationally-representative data in this study indicate that, even in a country with a high overall burden of asthma by world standards, Indigenous people still suffer disproportionately. Improved understanding of the distribution and determinants of asthma in the Indigenous population is an important step towards reducing the health disadvantage of Indigenous Australians.

## Competing interests

The author declares that she has no competing interests.

## Authors' contributions

As the sole author, JC contributed to the conception and design, analysis and interpretation of data, and to the drafting of the article and revising it critically for important intellectual content, and approved the final manuscript. No other individual has made a contribution to the manuscript that would warrant authorship.
